# Suppressors of Cytokine Signaling 3 Expression in Eosinophils: Regulation by PGE_2_ and Th2 Cytokines

**DOI:** 10.1155/2011/917015

**Published:** 2011-04-27

**Authors:** Esther López, María Paz Zafra, Beatriz Sastre, Cristina Gámez, Mar Fernández-Nieto, Joaquín Sastre, Carlos Lahoz, Santiago Quirce, Victoria Del Pozo

**Affiliations:** ^1^Immunology Department, IIS-Fundación Jiménez Díaz (IIS-FJD), Avenida Reyes Católicos 2, 28040 Madrid, Spain; ^2^CIBER of Respiratory Diseases (CIBERES) ISCII, Madrid, Spain; ^3^Allergy Department, IIS-Fundación Jiménez Díaz (IIS-FJD), 28040 Madrid, Spain; ^4^Allergy Department, Hospital Universitario La Paz, Madrid, Spain

## Abstract

Asthma and nonasthmatic eosinophilic bronchitis (NAEB) are respiratory disorders characterized by a predominance of Th2 cells and eosinophilic inflammation. Suppressors of cytokine signaling (SOCS) proteins play an important role in Th2-mediated allergic responses through control of the balance between Th1 and Th2 cells, particularly, SOCS3 and SOCS5. The aim of this study was to analyze SOCS expression in human peripheral blood eosinophils from patients with asthma, NAEB and healthy controls. SOCS expression in eosinophils from subjects was demonstrated by different techniques. Results showed that expression of SOCS3 in eosinophils and CD4 T cells from patients was higher than in healthy subjects. In addition, we demonstrated that prostaglandin E_2_ (PGE_2_) and Th2 cytokines are able to upregulate SOCS3 production in eosinophils and attenuate its degranulation. In conclusion, eosinophils are able to transcribe and translate SOCS3 protein and can contribute to the regulation of the Th1/Th2 balance through SOCS3 production.

## 1. Introduction

Th2 respiratory disorders, such as asthma, allergic rhinitis, and nonasthmatic eosinophilic bronchitis (NAEB), have been major public health problems in the last two decades. NAEB was originally described by Gibson et al. [1] and has subsequently been recognized as an important cause of chronic cough [2]. Asthma and NAEB are associated with a similar T-helper type 2 cytokine-driven airway inflammation [3, 4]. However, airway hyperresponsiveness and variable airflow obstruction, which are the hallmarks of asthma, are not present in NAEB.

Inflammatory mediators and cytokines play essential roles in the control of immune system; they not only act as growth factors, but also regulate the differentiation, maintenance, and activation of naïve effectors and the memory state of immune cells [5]. The Th1/Th2 balance determines the nature of an immune response [6]; however, the mechanism by which Th1 and Th2 cytokines cross-regulate the immune response remains unclear.

In both physiologic and pathologic conditions, cytokine function is strictly controlled. Cytokine signaling pathways are negatively regulated by the so-called suppressor of cytokine signaling (SOCS) family of proteins. There are eight members of the CIS-SOCS family [7]. Several reports have indicated that SOCS proteins are necessary for regulation of normal immune responses [8]. SOCS proteins not only act as simple negative-feedback regulators, but they are also involved in fine-tuning the immune response and in the crosstalk of complicated cytokine signal networks. Since cytokines are constantly present in the microenvironment of immune cells, signal regulation by SOCS-family proteins may be important for the proper progress, remission, and relapse of an immune response. Therefore, SOCS1, SOCS3, and SOCS5 participate in CD4+ Th-cell differentiation and in Th1/Th2-cell balance [9]. SOCS3 is predominantly expressed in Th2 cells and inhibits Th1 differentiation [10–12]. Conversely, SOCS5 is predominantly expressed in Th1 cells and inhibits Th2 differentiation [8, 13]. The cyclo-oxygenase product, prostaglandin E_2_ (PGE_2_), is produced by several cells in human airways, including the epithelium [14] and smooth muscle [15]; PGE_2_ is produced during inflammatory responses, and increased levels of PGE_2_ mediate some of the cardinal features of inflammation. In contrast, several studies suggest that in addition to its pro-inflammatory actions, PGE_2_ may also exert strong anti-inflammatory and bronchoprotective effects in patients with bronchial asthma [15–18]. Recently, it has been demonstrated that prostaglandins are capable of inducing SOCS3 expression [19–21]; moreover, we have reported that PGE_2_ is present in lung from subjects with NAEB and asthma [22], diseases that are characterized by high eosinophil counts. Thus, PGE_2_ may represent an endogenous protective mechanism in the airways as a modulator of immune responses. 

The SOCS implication in Th1/Th2 balance regulation and allergic phenotypes suggests a range of new therapeutic strategies that could reduce Th2-induced inflammation and eosinophilia. For these reasons, we determined if eosinophils, the characteristic inflammatory cells of asthma and NAEB, are able to express mRNA and synthesize SOCS3 protein,. In addition, we tested the hypothesis that both Th2 cytokines and PGE_2_ upregulate SOCS3 expression in eosinophils. This study reports SOCS3 production by eosinophils, a process which is regulated by cytokines and PGE_2_.

## 2. Materials and Methods

### 2.1. Subjects

Eight subjects with NAEB, 6 subjects with asthma, and 9 healthy control subjects were recruited from the Fundación Jimenez Díaz Allergy clinic outpatients and staff. The investigation has been conducted according to the principles expressed in the Declaration of Helsinki. The study was approved by the Ethical Committee from Fundación Jimenez Díaz, and informed consent of all participating subjects was obtained. Blood samples were obtained from adult donors. Total IgE levels were measured using the immunoCAP immunoassay system (Phadia, Uppsala, Sweden) and PGE_2_ levels by ELISA kit (Cayman Chemical Company, Ann Arbor, MI, USA).

Subjects with asthma had a consistent history of the disease and objective evidence of asthma (as defined by the American Thoracic Society) [23] for at least 6 months. These patients either showed a greater than 12% improvement in FEV_1_, 10 minutes after administration of 500 *μ*g of inhaled terbutaline, or had methacholine airway hyperresponsiveness (PC_20_ methacholine <16 mg/mL). Patients with asthma had mild persistent disease [24] and were clinically stable. None had a history of respiratory infection for at least the 6-week period preceding the study. We included both atopic and nonatopic patients in the asthmatic group, since no differences in the parameters assessed for both sets of patients had been observed previously. 

The subjects with NAEB had an isolated cough lasting >8 weeks, no symptoms suggesting variable airflow obstruction, normal spirometric values, a methacholine PC_20_ value >16 mg/mL, a normal chest radiograph, and sputum eosinophilia (sputum eosinophils >3%). 

For patients who were receiving inhaled corticosteroids, the drugs were withdrawn for at least 2 weeks before sputum induction. No patient was receiving oral corticosteroids (for at least 6 months prior to the study), leukotriene receptor antagonists, aspirin, or any other cyclooxygenase inhibitor.

### 2.2. Eosinophil and CD4 T Cell Purification

Eosinophils and CD4 T cells were purified from peripheral blood of healthy control and patient donors using a two-step procedure. First, the polymorphonuclear and mononuclear cell fractions were obtained by Ficoll gradient centrifugation. The second step involved removal of residual cells from the polymorphonuclear cell fraction. For eosinophil purification, CD2, CD3, CD14, CD16, CD19, CD20, CD36, CD56, CD123, and glycophorin A positive cells were discarded by a magnetic bead separation technique, as described in the manufacturer's procedure (EasySep; StemCell Technologies, Vancouver, Canada**).** The CD4 T cell purification from the mononuclear fraction was achieved using a similar protocol by removing monocytes, CD8 T cells, B cells and others by a magnetic bead separation technique, using CD14, CD16, CD19, CD20, CD36, CD56, CD123, TCR*γ*/*δ*, and glycophorin A antibodies. Remaining cells were CD4 T cells. The viability and purity of the cells were assessed by staining with trypan blue and flow cytometry, respectively. Eosinophils are defined as CCR3^+^CD16^−^cells, so we measured purity of eosinophils staining cells with fluorochrome-conjugated anti-CCR3 (CCR3-FITC) and anti-CD16 (CD16 PE) antibodies by flow cytometry. The viability and purity were routinely >98%.

### 2.3. Bronchial Biopsies

Bronchoscopies were performed (healthy controls *n* = 2, asthma *n* = 2, and NAEB *n* = 4) using a flexible fiberoptic or rigid bronchoscope. Biopsies were taken from the subcarinae of the left or right lower lobe using fenestrated forceps and finally immersed in Trizol reagent and frozen at −80°C until use.

### 2.4. Eosinophil Culture

Purified eosinophils from healthy control, asthmatics, and NAEB subjects were plated at 1 × 10^6^ cells/mL in RPMI 1640 with 10% fetal bovine serum, 100 U/mL penicillin, and 100 *μ*g/mL streptomycin at 37°C and 5% CO_2_. Cells were then treated with different doses of recombinant human IL-4 or IL-5 (0.1, 1, and 10 ng/mL; Bender MedSystem, Vienna, Austria) or IL-13 (0.5, 10 and 50 ng/mL; R&D System, MN, USA) or PGE_2_ (10^−4^ and 10^−6^ M; Cayman Chemical Company, Ann Arbor MI, USA) or IFN*γ* (5, 10, 20 ng/mL; R&D System, MN, USA) for different time periods (30–120 min). All conditions were performed in triplicate, in at least 4 independent experiments.

### 2.5. Confocal Microscopy

Purified eosinophils (5 × 10^5^ cells/mL; 100 *μ*L/slide) were adhered to slides by cytocentrifugation (Shandon Southern Instruments, Waltham, MA, USA). Adhered eosinophils were fixed and permeabilized with methanol and 0.1% Triton, respectively, and blocked with 4% BSA and 6% normal goat serum in PBS, washed, then incubated with SOCS3 antibody at 100 *μ*g/mL (Santa Cruz Biotechnology, Inc., Santa Cruz, CA, USA) or rabbit IgG as control, during 1 hour at room temperature. The slides were washed again and then exposed to Texas Red conjugated goat antirabbit IgG (Santa Cruz Biotechnology, Inc.) overnight at 4°C. The slides were observed by confocal microscopy (Leica Microsystems, Wetzlar, Germany). The fluorescence intensity over the area of a single cell was integrated, and nonspecific fluorescence (negative control) was subtracted. Net fluorescence intensity was divided by the average area of the eosinophil in *μ*m^2^ and expressed as fluorescence intensity units (FIU)/*μ*m^2^. For statistical analysis, average net intensity values were calculated from a minimum of 100 cells per sample.

### 2.6. Immunocytochemistry

Slides with purified eosinophils were treated with methanol and 3% hydrogen peroxide in PBS to block endogen peroxidase. Then, slides were blocked with 4% BSA and 6% normal goat serum in PBS, washed, and then exposed to SOCS3 antibody at 60 *μ*g/mL or rabbit IgG as control for 30 min at room temperature, and stained with the LSAB and System HRP Kit, according to the manufacturer's instructions (DAKO, Carpinteria, CA, USA). The slides were then lightly stained with hematoxylin and examined. 

### 2.7. Western Blot Analysis

The lysates from purified eosinophils (10 *μ*g total protein), and 5 *μ*g recombinant SOCS3 (rSOCS3) as positive control (Santa Cruz Biotechnology, Inc.), were resolved on SDS-PAGE and analyzed by Western blotting, using a 1 : 200 dilution of SOCS3 antibody or rabbit IgG as isotype control. The secondary antibody, HRP-conjugated goat antirabbit IgG (Santa Cruz Biotechnology, Inc.), was diluted 1 : 1000.

Chemiluminescent protein bands were detected by an ECL detection system (Amersham Biosciences, GE Healthcare, Buckinghamshire, UK) according to the manufacturer's protocol. The protein concentration was estimated according to the method of Bradford [25].

### 2.8. RNA Extraction and Real-Time Quantitative PCR of SOCS3 and SOCS5

Total RNA was extracted from eosinophils, CD4 T cells, and bronchial biopsies according to TRIzol reagent protocol (Invitrogen Life Technologies, CA, USA). Bronchial biopsies were homogenized previously. Two micrograms of RNA were DNase treated, followed by reverse transcription according to the kit instructions (Applied Biosystems, Warrington, UK). TaqMan PCR was performed using a 20 *μ*L final reaction volume containing 10 *μ*l of TaqMan Universal PCR Master Mix (Applied Biosystems, Branchburg, NJ, USA), 1 *μ*l of 20X Assays-on-Demand Gene Expression Assay Mix, and 9 *μ*l of cDNA diluted in RNase-free water. Each assay was performed in triplicate. The PCR conditions used in all reactions were 2 min at 50°C and 10 min at 95°C, with 40 two-step cycles (95°C for 15 s and 60°C for 60 s). Assays-on-Demand Gene Expression primers specific for SOCS3, SOCS5, and rRNA (used as an endogen) were obtained from Applied Biosystems (http://www.appliedbiosystems.com/). The genes analyzed in this study were examined for their relative expression by means of the ΔΔC_T_ method [26].

### 2.9. Eosinophil Degranulation

To determine the release of eosinophil peroxidase (EPO) from purified human eosinophils, cells were resuspended in assay buffer (PBS, 0.1% BSA, 10 nm HEPES, 10 nm Glucose; pH: 7.4) at 1 × 10^6^ cells/mL, mixed with cytochalasin B (10 *μ*g/mL), and 50-*μ*L aliquots were loaded into the wells of a 96-well microplate. Cells were stimulated with 20 *μ*L of C5a (300 nM) for 20 min at 37°C. Thereafter, 60 *μ*l of H_2_O_2_ (1 mM) were added to each well to start the peroxidase reaction. To detect the reaction, 70 *μ*l of 2.8 mM tetramethylbenzidine was used. Following incubation for 1 min at room temperature, the peroxidase reaction and the color development were stopped with 4 M acetic acid [27]. Microplates were analyzed on a bench reader at a wavelength of 630 nm. Data were expressed as the percentage of the maximal control response (C5a at 300 nM).

### 2.10. Statistical Analysis

The subject characteristics were described using descriptive statistics and expressed as geometric mean and standard deviation (SD), and median and range.

Results were compared and evaluated using the Kruskal Wallis test and posttest Dun's multiple comparison tests. Correlation coefficients and statistical significance were determined using Spearman correlation coefficient. Statistical significance was recognized at *P* ≤ .05. Statistical analyses were performed using GraphPad InStat3 (GraphPad Software Inc., San Diego, CA, USA).

## 3. Results

### 3.1. Clinical Characteristics of the Subjects

Eight subjects with NAEB, six subjects with asthma, and nine healthy control subjects were recruited from the Fundación Jimenez Díaz Allergy Clinic outpatients and staff. The subjects' clinical characteristics are shown in Table 1. 

The age of asthma and NAEB patients was very similar (41.75 ± 8.94 and 38.55 ± 12.06 years, resp.), and no differences existed in the evaluated parameters as a function of age or gender in adult patients with asthma or NAEB.

Predicted values for FEV_1_ fell within a normal range, although asthmatic patients showed a significant decrease compared to healthy control subjects and NAEB patients (*P* < .05). The median percentage of eosinophils on whole blood was significantly higher in the subjects with NAEB and asthma than in healthy control subjects (4.4%, 2.66%, and 0.14%, resp.; *P* < .01 and *P* < .05). In concordance with these data, sputum eosinophils were also statistically higher in NAEB and asthma patients than in healthy controls (15.9%, 15.1%, and 2.1% resp.). No significant differences were observed in the eosinophil count between patients with asthma and patients with NAEB, as shown in Table 1. The IgE level in patients with NAEB and asthma was significantly higher than in healthy controls (*P* < .05). There was a tendency toward higher levels of PGE_2_ in patients with NAEB (*P* < .05) and asthma (not significant).

### 3.2. SOCS Gene Expression Correlates with Th2 Respiratory Disorders

Asthma and NAEB are allergic diseases characterized by massive infiltration of eosinophils and T cells secreting Th2 cytokines into the pathologic site. We therefore examined whether purified CD4 T cells (>98%) from patients with NAEB and asthma had higher levels of SOCS3 gene expression as consequence of the preferential development of Th2 cells. Expressions of SOCS3 and SOCS5 mRNA were analyzed by real-time quantitative PCR in peripheral CD4 T cells from adult patients with NAEB, asthma and healthy controls.

**Figure 1:**
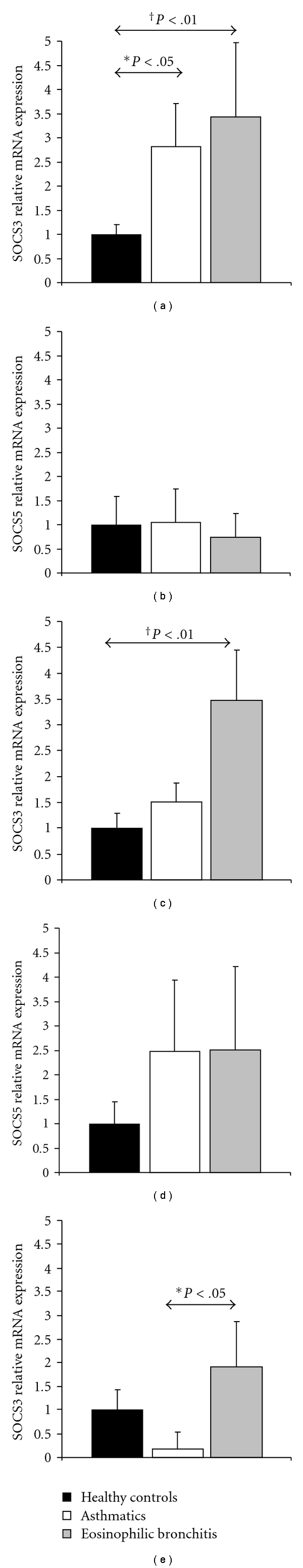
Semiquantitative expression of SOCS3 and SOCS5 genes in peripheral blood purified CD4 T cells and eosinophils. Relative mRNA levels of SOCS3 and SOCS5 in CD4 T cells ((a) and (b)), and eosinophils ((c) and (d)) of healthy controls, asthmatic and NAEB patients were determined by real-time quantitative PCR. Values were normalized with rRNA gene used as an endogen. Black boxes represent the healthy control group (mean ± SD, *n* = 9), white boxes represent the asthmatic patient group (mean ± SD, *n* = 6), and lineated boxes represent the NAEB group (mean ± SD, *n* = 8). Significant differences in the levels of SOCS3 expression in CD4 T cells were obtained for asthmatic and NAEB patients versus the healthy control group (*P < .05 and **P < 0.01, resp.). In eosinophils, the SOCS3 level was significantly higher in NAEB patients than in healthy controls (**P < .01). In the case of SOCS5, no significant differences were found between the groups. (e) SOCS3 mRNA levels in bronchial biopsies from healthy controls (mean ± SD, *n* = 2), asthmatics (mean ± SD, *n* = 2), and NAEB patients (mean ± SD, *n* = 4). Significant differences in the levels of SOCS3 expression were obtained for asthmatic versus NAEB patients (*P < .05). The results represent relative gene expression, as determined by the ΔΔC_T_ method.

The expression of SOCS3 mRNA in patients with asthma and NAEB was significantly higher than in healthy individuals (*P* < .05 and *P* < .01, resp.; Figure 1(a)); however, no significant difference was observed in the expression of SOCS5 between patients and controls (Figure 1(b)).

### 3.3. Eosinophils from Donors with Th2 Respiratory Disorders Exhibit Increased SOCS3 mRNA Expression in Comparison to Normal Eosinophils

Because the accumulation of eosinophils is a feature of both asthma and NAEB, we determined if eosinophils are able to transcribe and translate mRNA for SOCS3. Thus, SOCS mRNA expression in purified eosinophils (>98%) from patients and healthy individuals was studied. SOCS3 mRNA levels in patients with NAEB were higher than in healthy controls (*P* < .01, Figure 1(c)); although there was a weak SOCS3 level increase in patients with asthma (1.51-fold), it did not reach statistical signification versus healthy subjects. SOCS5 expression was higher in eosinophils from patients, but there were no significant differences between the patient groups and control subjects (Figure 1(d)). As elevation of serum IgE is a characteristic of patients with NAEB and asthma, we determined the correlation between serum IgE and the expression of SOCS3. Individuals with a high expression of SOCS3 had high levels of serum IgE (*P* < 03, *r*: 0.5; data not shown).

### 3.4. Differential Expression of SOCS3 mRNA between Bronchial Biopsies from Asthmatics and NAEB Patients

The lung undergoes dramatic changes in asthma and NAEB. Because SOCS3 is involved in the regulation of the Th1/Th2 axis in allergic diseases, we measured the SOCS3 mRNA expression in bronchial biopsies from healthy controls, asthmatics, and NAEB subjects (Figure 1(e)). 

 Our results showed that SOCS3 mRNA expression in bronchial biopsies was strikingly increased (12.7-fold higher) in subjects with NAEB as compared with asthmatics (*P* < .05).

### 3.5. Identification of SOCS3 in Eosinophils by Immunocytochemistry, Confocal Microscopy, and Immunoblotting

**Figure 2:**
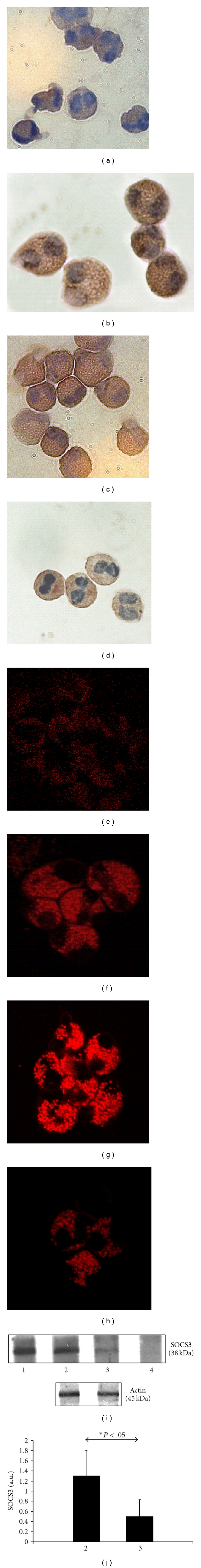
SOCS3 expression in peripheral blood eosinophils from Th2 patients analyzed by immunohistochemical, immunofluorescence, and Western blot techniques. Eosinophils from asthmatic and NAEB patients within healthy controls were adhered to slides and incubated with peroxidase-conjugated goat antirabbit IgG against SOCS3 antibody ((b), (c), and (d)) or rabbit IgG as a control (a), and Texas Red conjugated goat antirabbit IgG against SOCS3 antibody ((f), (g), and (h)) or rabbit IgG as a control (e). The slides were observed by optical ((a), (b), (c), (d)) or confocal ((e), (f), (g) and (h)) microscopy. Western blot analysis of the cytosolic extract of purified eosinophils was achieved using antibody against SOCS3 (i). Lane 1: recombinant SOCS3 was loaded as a positive control; lane 2: eosinophil lysate from NAEB patients; lane 3: eosinophil lysate from healthy control patients; lane 4: isotype negative control. The picture is a representative example of 5 individuals, all displaying similar results. (j): SOCS3 bands were quantified by densitometry and corrected by actin expression; data are expressed as the mean ± SD, *n* = 5, *P < .05 (2: NAEB; 3: healthy patients).

Immunocytochemistry, confocal microscopy, and immunoblotting were performed to confirm the expression of SOCS3 in eosinophils.  Figure 2 shows an example of highly purified eosinophil preparations from asthmatics (Figures 2(b) and 2(f)), NAEB (Figures 2(c) and 2(g)) and healthy subjects (Figures 2(d) and 2(h)), in which eosinophils clearly show the characteristic bilobular nuclei of mature blood eosinophils. There were eosinophils positively immunostained for SOCS3 in asthmatics and NAEB samples, as indicated by the expression of the brown reaction product (Figures 2(b) and 2(c), resp.) or red fluorescence (Figures 2(d) and 2(h), resp.). The expression of SOCS3 protein measured as fluorescence intensity was higher in patients with NAEB (30.56 ± 8.81 FIU/*μ*m^2^; Figure 2(g)) than in asthmatics (12.35 ± 5.5 FIU/*μ*m^2^; Figure 2(f)) whereas in healthy controls was hardly intense (5.765 ± 2.64 FIU/*μ*m^2^ Figure 2(h)). Similar results were obtained by immunocytochemistry (Figures 2(c) and 2(b)).

 When we examined the distribution of SOCS3 in eosinophils, using both techniques we showed that SOCS3 immunoreactivity or immunofluorescence was largely confined to the eosinophil cytoplasmic compartment with granular expression pattern (Figures 2(b), 2(c), 2(f), and 2(g)). The negative control was treated with HRPO isotype antibody, and the absence of specific staining is revealed Figure 2(a). Also, Figure 2(e) represents images taken from Texas Red isotype control, and demonstrates that there was negligible autofluorescence or nonspecific binding in these samples.

Finally, SOCS3 protein production by eosinophils was verified by Western blot, using SOCS3 antibody (Figure 2(i)). The result pointed out a protein band with a similar molecular weight to the commercial positive control (rSOCS3, 38 kDa) in the protein lysates of highly purified eosinophils from patients with NAEB and control subjects. The SOCS3 levels were quantified by densitometry and normalized to actin levels (Figure 2(j)), and the data demonstrated that the quantified band was statistically more intense in eosinophils from patients compared to healthy individuals (*P* < .05). This data confirm the SOCS3 protein expression in eosinophils.  Figure 2 shows a representative example of 5 individuals, all of whom display similar results.

### 3.6. Th2 but Not Th1 Cytokines Stimulate SOCS-3 mRNA Expression in Eosinophils

The increased expression of SOCS3 in eosinophils from patients with NAEB and asthma compared with controls is probably due to factors that could regulate SOCS3 expression in these cells under disease conditions. For this reason, *in vitro* effects of Th2 and Th1 cytokines in SOCS3 mRNA expression in eosinophils were assessed using real-time quantitative PCR.

**Figure 3:**
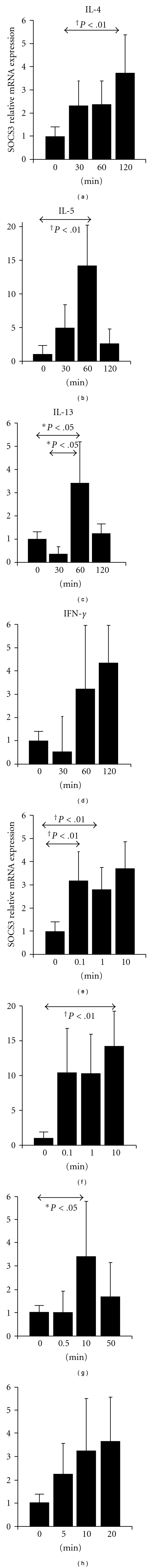
SOCS3 expression in peripheral blood eosinophils treated with Th2 cytokines and IFN-*γ*. Purified eosinophils from healthy donors were cultured with 10 ng/mL of IL-4 (a), IL-5 (b), IL-13 (c), or IFN-*γ* (d) for different periods of time. Dose-response curves were performed with IL-4, IL-5, IL-13, or IFN-*γ* (e)–(h) at maximal time response; SOCS3 mRNA was measured by real-time quantitative PCR. The results are expressed as a fold induction relative to untreated eosinophils, and significant differences are indicated. Data represent the geometric mean ± SD from four individuals, and each measure was performed in triplicate.

The treatment of eosinophils from healthy subjects with recombinant human IL-4, IL-5, or IL-13 (Th2 cytokines) induces upregulation of SOCS3 mRNA expression in a time-dependent manner, with higher levels of SOCS3 mRNA at 120 min for IL-4 (Figure 3(a)) and at 60 min for IL-5, and IL-13 (Figures 3(b) and 3(c)). Furthermore, SOCS3 mRNA expression was significantly enhanced by the incubation with human recombinant IL-4, IL-5, or IL-13 in a dose-dependent manner (Figures 3(e), 3(f), and 3(g)). Our results showed that SOCS3 production was strikingly increased (14.10-fold; Figure 3(f)) in IL-5-induced eosinophils as compared with IL-4-, or IL-13-induced eosinophils (3.71-fold; Figure 3(e) and 3.39-fold; Figure 3(g), resp.). In contrast, when eosinophils were cultured with IFN-*γ*, typically secreted by Th1 cells, SOCS3 expression was not statistically affected at any dose or time assayed (Figures 3(d) and 3(h)). Similar results came out when stimulation was carried out with IL-2, another well-known Th1 cytokine (data not shown). Collectively, these results demonstrate that SOCS3 expression is activated in response to increased amounts of Th2 cytokines.

**Figure 4:**
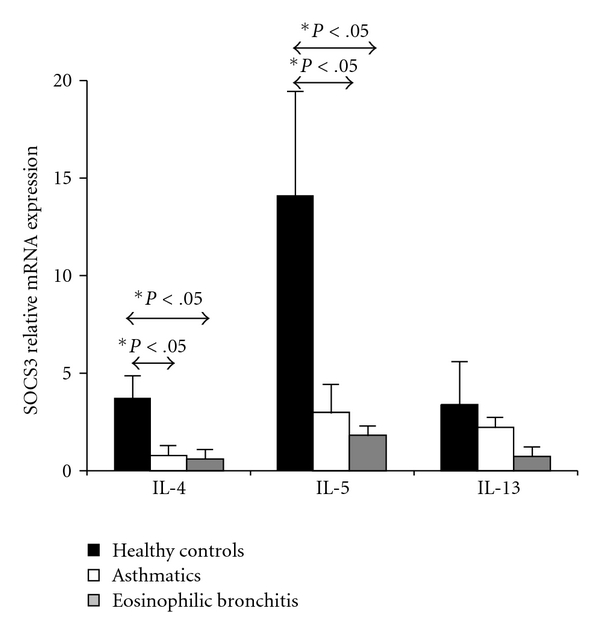
SOCS3 mRNA expression in Th2-stimulated eosinophils from healthy controls, asthmatics, and NAEB patients. Eosinophils from healthy controls, asthmatics, and NAEB patients were cultured with 10 ng/mL of IL-4 or IL-5, or IL-13 during 60 min. SOCS3 mRNA was measured by real-time quantitative PCR. The results are expressed as a fold induction relative to untreated eosinophils, and significant differences are indicated. Data represent the geometric mean ± SD from three individuals, and each measure was performed in triplicate.

We wanted to compare the effects of Th2 cytokines on the expression of SOCS3 mRNA in eosinophils from healthy controls, asthmatics, and NAEB patients. When eosinophils from asthmatics and NAEB patients were treated during optimal period of time with IL-4, the relative level of SOCS3 mRNA expression was significantly lower than in healthy controls with the same treatment (0.8 ± 0.3, 0.6 ± 0.6, and 3.71 ± 1.2 resp., *P* < .05, Figure 4). Similar results were obtained when eosinophils were cultured with IL-5 (3.02 ± 2.2, 1.86 ± 0.6 and 14.11 ± 4.2, resp., *P* < .05, Figure 4). However, stimulation with IL-13 did not significantly modify SOCS3 mRNA expression.

### 3.7. PGE_2_ Treatment Produces Increased SOCS3 mRNA Expression in Eosinophil Cultures

**Figure 5:**
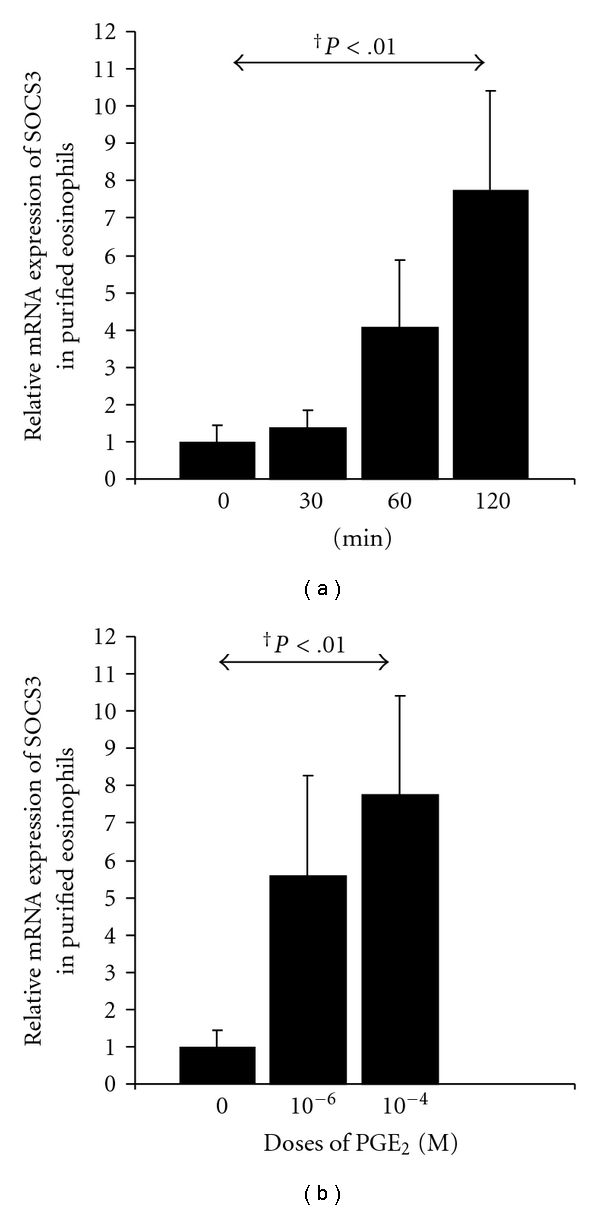
PGE_2_ stimulation of SOCS3 mRNA expression. Purified eosinophils from healthy donors were cultured with 10^−4^ M of PGE_2_ for up to 120 min (a), and with increasing doses of PGE_2 _at 120 min (b), and SOCS3 mRNA was measured by real-time quantitative PCR. The results are expressed as a fold induction relative to untreated eosinophils, and significant differences are indicated. Data represent the geometric mean ± SD from four individuals, and each measure was performed in triplicate.

Because it has been reported that PGE_2_ upregulates SOCS3 gene expression in different cell types, we examined the effect of PGE_2_ on SOCS3 expression in eosinophils from healthy subjects. We tested different doses of PGE_2_ (10^−4^ and 10^−6^ M), and SOCS3 gene activation was determined by real-time quantitative PCR. After treatment of eosinophils with PGE_2_, SOCS3 mRNA expression was upregulated at 60 and 120 min (Figure 5(a)). This regulation was dose dependent, and significant differences were found compared with the control eosinophils (Figure 5(b)). PGE_2, _10^−4^ M and 10^−6^ M, increased SOCS3 gene expression by 7.76- and 5.58-fold, respectively.

These data indicate that PGE_2_ induces SOCS3 mRNA expression, suggesting that PGE_2_ can be a regulatory metabolite of SOCS3 protein production in eosinophils.

### 3.8. Th2 Cytokines and PGE_2_ Inhibit Eosinophil Degranulation

**Figure 6:**
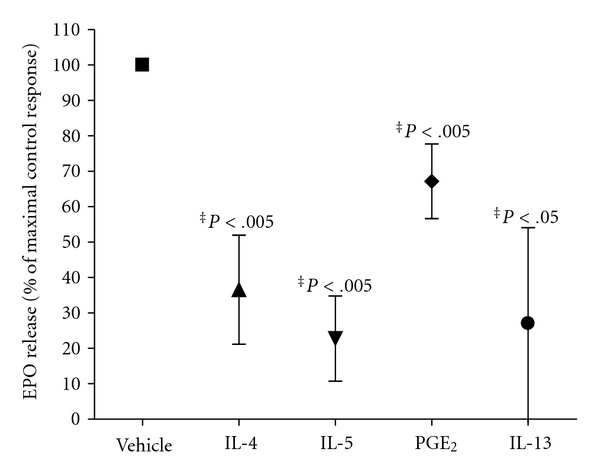
Th2 cytokines and PGE_2_ inhibit eosinophil degranulation. Purified human eosinophils were pretreated with vehicle, IL-4 (10 ng/mL), IL-5 (10 ng/mL), IL-13 (10 ng/mL), and PGE_2_ (10^−6^ M) for 1 or 2 hours and then stimulated with C5a (300 nM) for 30 min at 37°C. The release of EPO activity into supernatants was determined by photometry. Data were expressed as a percentage of the maximal control response (300 nM) and are shown as the mean ± SD; *n* = 5–9; **P < .005, *P < .05 versus C5a alone (vehicle).

We assessed the biological significance of SOCS3 augmentation in eosinophils by examining the effects of Th2 cytokines and PGE_2_ in eosinophil degranulation. We observed that C5a-induced release of EPO was significantly attenuated by both Th2 cytokines and PGE_2_ (Figure 6). All inhibitions obtained were statistically significant and is ranged between 32.5% and 77%, obtained with PGE_2_ and IL-5, respectively.

## 4. Discussion

Our results reveal that eosinophils are able to transcribe and express SOCS3 at the protein level, and this production is upregulated by IL-4, IL-5, IL-13, and PGE_2_. In addition, SOCS3 production was increased in patients with NAEB. These data suggest that eosinophils can contribute to the regulation of the inflammatory response through SOCS3 production. 

This study examined SOCS protein expression in blood eosinophils from patients with two Th2 conditions (asthma and NAEB). These respiratory disorders are characterized by the recruitment and activation of inflammatory cells, including eosinophils and lymphocytes, which produce cytokines [22]. However, the amplitude and duration of the response depend on precise fine-tuning and coordination of immune cell responses, and these aspects are regulated by endogenous feedback regulators of cytokine activities. Prominent among this class of intracellular regulators are members of the SOCS proteins family [5, 7]. Significant interest in the SOCS family stems from the belief that SOCS proteins may integrate multiple cytokine signals and mediate cross-communication between antagonistic cytokines elaborated by different cells through inhibitory effects on cytokine receptors and signaling molecules. 

SOCS proteins exert negative regulation of cytokine signaling in a variety of ways and are known to be involved in the pathogenesis of many inflammatory diseases. Hence, some members of the SOCS protein family, and SOCS1, SOCS3, and SOCS5 in particular, participate in regulation of the Th1/Th2 balance [28, 29]. 

According to this, the evaluation of SOCS3 in bronchial biopsies from healthy controls, asthmatics, and NAEB subjects ascertained the expression at mRNA level of this negative regulator. Notably, this expression was enhanced in bronchial biopsies from NAEB subjects in comparison with those from healthy control or asthmatics. 

Considering that eosinophils play a prominent pro-inflammatory role in allergic airway inflammation, and noting that other authors have recently described the SOCS3 expression in different cellular types, such as macrophages and microglia [30], we determined if blood eosinophils are able to express SOCS3 and SOCS5 at the protein level and thus contribute to regulate cytokine balance in NAEB and asthma, conditions in which eosinophils are one of the most important cell types. 

We have demonstrated that blood eosinophils express SOCS proteins. SOCS3 and SOCS5 gene expressions were detected by real-time quantitative PCR, and SOCS3 protein was detected in the cytosol of blood eosinophils with granular expression pattern by immunohistochemistry, immunofluorescence, and immunoblotting.

Moreover, blood eosinophils from patients with Th2 disorders exhibit higher levels of SOCS3 mRNA than healthy subjects. These data suggest that eosinophils may play an important role in regulating inflammation in respiratory disorders through SOCS3 expression. We tested the effects of Th2 cytokines (IL-4, IL-5, and IL-13) on the expression of SOCS3 in blood eosinophils *in vitro*, and we showed upregulation of SOCS3 expression in a time- and dose-dependent manner, thus corroborating SOCS-3 activation in response to the Th2 environment under disease conditions. We also found that SOCS-3 expression in blood eosinophils from patients with asthma and NAEB was correlated with serum IgE levels (*r* = 0.5, *P* < .03). This result can be since both phenomenons are occurring simultaneously after IL-4 stimulation. The increase of SOCS3 expression in response to Th2 cytokines in healthy individuals is markedly greater than in those from asthmatic and NAEB patients, indicating that eosinophils from individuals with asthma and NAEB had impaired upregulation of SOCS3 after Th2 stimulation. These results may be explained because, in these patients, Th2 cytokines are present in the microenvironment conferring an intrinsic Th2 status that could provide a negative feedback mechanism, so further stimulation hardly causes additional effect in SOCS3 mRNA expression. Similar findings have been published [31, 32]. 

 It was also interesting to evaluate the potential role of IFN-*γ* in regulating SOCS3 expression in purified eosinophils due to its ability to counteract the effects of Th2 cytokines in various cells types, including T cells, B cells, endothelium, and epithelium [33]. Nevertheless, our results did not reveal any variation in the SOCS3 mRNA expression, suggesting that SOCS3 is not regulated by this mediator. 

We also analyzed the expression of SOCS3 and SOCS5 in peripheral blood CD4 T cells from patients with NAEB and asthmatics compared with healthy controls. The results showed that the expression of SOCS3 in patients was significantly higher than in control subjects, and we found a correlation between level of SOCS3 mRNA in CD4+ cells and IgE level (*r* = 0.58, *P* < .04). Similarly, Seki et al. [32] reported that the level of SOCS3 mRNA in peripheral CD3+ cells was elevated and correlated with the serum IgE level in patients with atopic dermatitis and asthma. Analysis of the levels of SOCS5 gene expression indicated that there was no significant change between the groups. These results are in concordance with other studies in which there were no differences in SOCS5 gene expression level between healthy subjects and those with asthma or atopic dermatitis [34, 35].

SOCS3 has the potential to modify responsiveness of airways to important cytokines. SOCS3 is expressed in eosinophils, and although cytokines themselves are likely to contribute to this, we have shown that PGE_2_, which is produced in airways, can also stimulate SOCS3 expression in eosinophils. Several studies have reported that PGE_2_ treatment not only stimulates the expression of SOCS3 in different cells, but also prolongs the stability of SOCS3 mRNA [19–21, 36]. Recently, we have described increased PGE_2_ levels in the airways of patients with NAEB [16], so it is not surprising that individuals with NAEB who have high PGE_2_ levels also have high levels of expression of SOCS3 (*r* = 0.81, *P* < .05). When we analyzed the effect of PGE_2_ in SOCS3 expression in blood eosinophils *in vitro*, we observed a significant increase in SOCS3 mRNA expression in a time- and dose-dependent manner. 

The differential regulation of SOCS3 expression by distinct stimuli creates a situation whereby potentiation of SOCS3 expression is possible. In our study, we observed that IL-4, IL-5, and IL-13_,_ at doses to produce maximal SOCS3 expression, inhibited eosinophil degranulation, probably through SOCS3 augmentation. According with these results, Hebenstreit et al. show that SOCS1 and SOCS3 can negatively regulate IL-4- and IL-13-induced CCL26 expression [37], which is one of the main attractants of eosinophils. Both results highlight an important mechanism SOCS3 mediated for regulating the activation and recruitment of eosinophils during inflammation by limiting the effects of Th2 cytokines. 

Also, in our study eosinophils degranulation is partially inhibited by PGE_2_. Perhaps, in addition to other effects, PGE_2_-induced SOCS3 expression may be important in regulating responsiveness to cytokines and could be involved in the pathogenesis and regulation of Th2 respiratory disorders. Recently, Sturm et al. point out that prostaglandin E2 inhibits eosinophil trafficking through EP2 receptors [38]. This data provide a novel mechanistic concept to substantiate previous observations that PGE_2_ acts as an anti-inflammatory mediator in human airways [15–18].

The diverse and specific effects of PGE_2_ depend on different EP receptor subtypes. In eosinophils, all forms of EP receptor genes are expressed [39]; in our patients, EP_2_ and EP_4_ were expressed at a higher level when compared with EP_1_ and EP_3 _(data not shown)_, _so these results suggest that PGE_2_-induced modulation of cytokines signaling can occur via EP_2_ and/or EP_4_ receptors, although further studies are necessary to confirm this notion.

## 5. Conclusions

The most important finding in the current study was that expression of SOCS3 protein has been described for the first time in eosinophils, and as SOCS3 has an important role in regulating the onset and maintenance of Th2-mediated allergic immune diseases, this could contribute to improve the understanding of the pathways implicated in the regulation of these diseases. It is also a novel finding that PGE_2, _which is highly produced in some Th2 diseases_, _can modulate effects on the immune response via regulation of cytokine signaling.

A precise regulation of both, the magnitude and duration of cytokine signaling, is essential for orchestration of immune reactions. Interventions that regulate Th2 cytokine effector pathways are attractive as potential therapeutic targets. The implication of SOCS proteins in the regulation of the Th1/Th2 balance suggests a range of new therapeutic strategies that might reduce Th2-induced inflammation and its consequences in eosinophilia.

## Figures and Tables

**Table 1:** Clinical characteristics of study subjects.

	Healthy controls	Nonasthmatic eosinophilic bronchitis (NAEB)	Asthma
*N*	9	8	6

Age (years)	23 (20–30)	40 (31–55)	37(21–61)
Male (%)	4 (44.4%)	4 (50%)	3 (50%)
Atopy (%)	0	5 (62.5%)	3 (50%)
FEV_1_ predicted^a^	105 (100–129)	108.95 (96–115)^§^	99,47 (73–110)*
FEV_1_/FVC^a^	85 (79–92)	80.6 (74.5–89.6)	78,91 (77.6–95.2)
Total IgE (kU/L)^a^	21.15 (4.52–144)	163 (34.50–605)*	65.2 (57.6–360)*
Eosinophil count (%)	0.14 (0–3.32)	4.40 (1.5–8.39)^†^	2.66 (1.34–7.92)*
Sputum eosinophils (%)^a^	2.1 (0.1–9.7)	15.9 (2–35)	15.1 (0.6–52.3)
PGE_2_ in sputum supernatant (pg/mL)^a^	2.89 (0.78–5.28)	15.582 (3.81–1336)*	33.8 (12.9–54.7)

FVC: forced vital capacity, FEV1: forced expiratory volume in the first second.

^a^Median (range).

*P < .05 (versus healthy control; Kruskal Wallis test; posttest Dunn's multiple comparison test).

^†^ *P* < .01(versus healthy control; Kruskal Wallis test; post test Dunn's multiple comparison test).

^§^ *P* < .05 (versus asthma patients; Kruskal Wallis test; post test Dunn's multiple comparison test).
